# Maternal Perception of Decreased Fetal Movement in One Twin: A Clue Leading to the Early Detection of Absent Variability due to Acute Twin-to-Twin Transfusion Syndrome

**DOI:** 10.1155/2013/345808

**Published:** 2013-07-25

**Authors:** Hirotada Suzuki, Tomoyuki Kuwata, Akihide Ohkuchi, Yukari Yada, Shigeki Matsubara, Mitsuaki Suzuki

**Affiliations:** ^1^Department of Obstetrics and Gynecology, Jichi Medical University School of Medicine, 3311-1 Yakushiji, Shimotsuke-shi, Tochigi 329-0498, Japan; ^2^Department of Pediatrics, Jichi Medical University School of Medicine, 3311-1 Yakushiji, Shimotsuke-shi, Tochigi 329-0498, Japan

## Abstract

Decreased fetal movement (DFM) perceived by pregnant women sometimes indicates imminent fetal jeopardy. It is unknown whether this also holds true for twin pregnancy. A 27-year-old primiparous woman with monochorionic diamniotic (MD) pregnancy had a slight difference of amniotic fluid volume at 31^2/7^ weeks of gestation. DFM only in one twin at 31^4/7^ weeks of gestation prompted her to receive urgent consultation. Since cardiotocogram indicated absent variability of one twin, we performed Cesarean section. Male infants weighing 2060 g and 1578 g were delivered; hemoglobin was 20.7 versus 10.8 g/dL, respectively; cardiothoracic ratio was 70% versus 44%, respectively, indicating acute twin-to-twin transfusion syndrome (TTTS). The recipient infant had heart failure, which was still observed at 1 month postpartum. In conclusion, maternal perception of DFM indicated imminent fetal death or jeopardy caused by acute TTTS, suggesting that education regarding DFM for women with twin pregnancy may be clinically important.

## 1. Introduction

Decreased fetal movement (DFM) perceived by the mother sometimes indicates imminent fetal death or jeopardy in singleton pregnancy [1–5]. Does this also hold true for twin pregnancy? There has been no report that maternal perception of DFM in twin pregnancy is associated with imminent fetal death or jeopardy. We here describe a woman with twin pregnancy in whom DFM of one twin prompted her to receive urgent consultation to us, resulting in early detection of absent variability caused by acute twin-to-twin transfusion syndrome (TTTS).

## 2. Case Presentation

**Figure 1:**
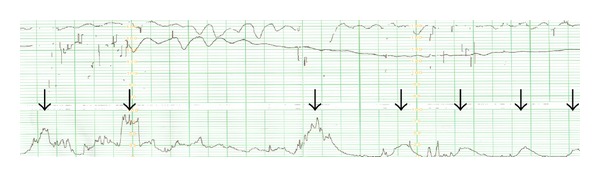
Cardiotocogram indicated absent variability in the right twin (I), and reassuring pattern in the other left twin (II), showing uterine contractions every one to three minutes. Arrows indicate uterine contractions.

**Figure 2:**
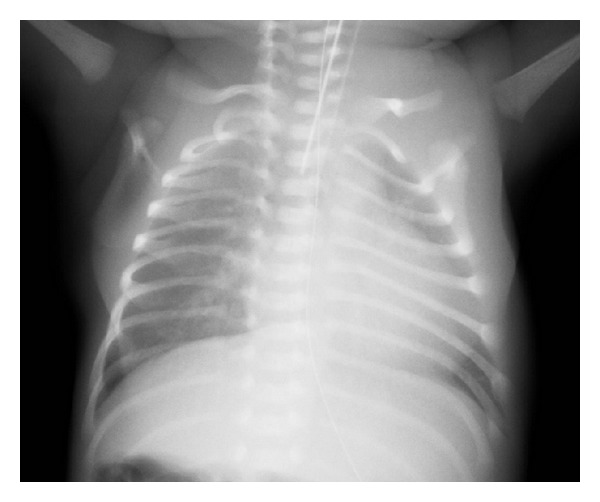
The chest X-ray of the first recipient-twin showed cardiac dilatation: the cardiothoracic ratio was 70%.

A 27-year-old primiparous woman with monochorionic diamniotic (MD) twins conceived spontaneously, sought maternal check-up at 9 weeks of gestation in a primary care hospital, and was checked every two weeks. At 26^3/7^ weeks of gestation, she was referred to our tertiary center due to “Satogaeri bunben” (a traditional ritual (support system) for perinatal women in Japan; “Satogaeri” means returning to the original family town or house and “bunben” means delivery); and her pregnant course every two weeks was uneventful until 31^2/7^ weeks, when the MD twins with vertex (I: right-lower side)-vertex (II: left-upper side) presentation showed amniotic fluid pocket of 7.9 and 2.4 cm, respectively, indicating the appearance of slight difference. Estimated fetal body weight in the first and second twins was 1683 g and 1531 g, respectively, suggesting that there was not significant fetal body weight discordancy. Both twins showed reassuring fetal statuses and water-filled bladders. Although this condition did not meet the criteria of TTTS [6], we planned to admit her 7 days later to monitor the fetuses more closely because amniotic fluid imbalance emerged abruptly. We educated her regarding DFM as a sign of imminent fetal death or jeopardy; therefore, she deliberately started to perceive fetal movements of both twins everyday [7]. Fortunately, she could perceive fetal movement in a fetus-by-fetus manner; she could easily discern one fetal movement from the other fetal movement because she had always felt the two fetuses' movements in almost the same location. Thus, although we did not recommend “modified count to 10” method in a fetal movement count chart [7] due to twin pregnancy, she has been paying attention to fetal movements of both fetuses. Five days before the booking day, she perceived DFM only in the right twin (I), with the left cotwin (II) moving as usual. In addition, she felt frequent uterine contractions. These changes prompted her to receive urgent consultation to us. Then, cardiotocogram indicated absent variability in the right twin (I), while reassuring pattern in the left twin (II), with uterine contraction every one to three minutes (Figure 1). There were no ultrasonographic signs indicating placental abruption. Considering immature cervix and acute appearance of absent variability of one twin, we decided to perform Cesarean section under general anesthesia. The first twin (I) was male infant weighing 2060 g, with Apgar score of 1 and 5 at 1 and 5 minutes, respectively; and blood gas analysis in the umbilical artery showed pH of 7.13 and base excess of −10.8. The second twin (II) was male infant weighing 1578 g, with Apgar score of 1 and 6 at 1 and 5 minutes, respectively; and blood gas analysis in the umbilical artery showed pH of 7.20 and base excess of −10.8. Hemoglobin of the first and second twins 2 hours after delivery was 20.7 and 10.8 g/dL, respectively; brain natriuretic peptide of each twin 2 days after delivery was 1459 and 2027 pg/mL (normal range: <18.4 pg/mL), respectively; and cardiothoracic ratio of each twin was 70% (Figure 2) and 44%, respectively. The first twin showed high systolic blood pressure (80–100 mm Hg) and polyuria (4–6 mL/kg/hour) just after delivery; left ventricular dimension was thick, ejection fraction was 65%, and mild tricuspid regurgitation and mitral regurgitation were observed; and he had generalized edema just after delivery, but body weight decreased 20% during 8 days after delivery. In contrast, the second twin showed very low systolic blood pressure (20–30 mm Hg) and collapsed inferior vena cava just after delivery, indicating hypovolemia; however, echocardiography did not show any significant findings. Then, we diagnosed this condition as acute TTTS in view of both pregnancy course and the fetal findings. While the first recipient-twin was responded by diuretics and had large amount of urination, intraventricular hemorrhage and ventriculomegaly occurred 2 days after delivery; the ventriculomegaly was still observed at 1 month postpartum. The second donor twin showed no sequelae at 1 month postpartum. Patient anonymity was preserved and informed consent for reporting was obtained from the parents.

## 3. Discussion

Although it is not yet determined whether recommending all pregnant women to count fetal movements (universal fetal movement counting) reduces the fetal death, obstetricians should ask a pregnant woman to consult the caregivers when she felt DFM [8]. This case dramatically illustrated that maternal perception of DFM may save fetal/infant lives in not only singleton pregnancy but also in twin pregnancy, indicating the importance of educating pregnant women regarding DFM. To our knowledge, no previous reports showed possible effectiveness of educating pregnant women with twin pregnancy regarding DFM.

In this pregnant woman with MD twins, fetal movement in one twin acutely decreased and regular uterine contraction occurred at the same time. Although apparent placental vascular anastomoses were not determined by visual inspection in this case, previous reports clearly indicated that placenta in almost all MD twins has vascular anastomoses [9–11]. We do not know the reasons why DFM and frequent uterine contractions occurred simultaneously; however, uterine contraction may have increased the flow between the fetuses, which might have triggered, or might have led to, acute TTTS [12, 13].

It may have been more cautious if we admitted her at the time when amniotic fluid imbalance emerged abruptly at 31^2/7^ weeks of gestation, even though the criterion of TTTS was not fulfilled then. Van Mieghem et al. [14] showed that a fluid discordance of ≥3.1 cm diagnosed before 20 weeks selected the group at highest risk for TTTS. However, since a predictor of acute TTTS has not been identified yet, it is very difficult to predict acute TTTS, and we have not yet had a definite measure for it. Furthermore, we do not know if earlier admission might have led to earlier detection of occurrence of acute TTTS in our case with MD twins showing slight amniotic fluid imbalance after 26 weeks of gestation, and thus to earlier intervention before establishing heart failure in the first recipient twin. 

When the fetal position in twin pregnancy frequently changes, the pregnant woman may feel difficulty in discriminating the movements in a fetus-by-fetus manner in other words, it may be very difficult for her to know one's decreased activity if only one twin becomes almost inactive. However, we believe that educating pregnant women with twin pregnancy regarding clinical meaning of DFM may be better encouraged even for women with twin pregnancy because we, from our experiences on twin pregnancy, know that not a few women with twin pregnancy could perceive fetal movements in a fetus-by-fetus manner. At least, letting them notice the importance of DFM may be far better than letting them unnoticed it.

We here describe the maternal perception of DFM in one twin indicating fetal jeopardy caused by acute TTTS. The maternal perception of DFM even in twin pregnancy may be associated with imminent fetal death. Educating pregnant women with twin pregnancy regarding clinical meaning of DFM may be clinically important for saving fetal/infant lives.
